# Prediction of programmed cell death protein 1 in hepatocellular carcinoma patients using radiomics analysis with radiofrequency-based ultrasound multifeature maps

**DOI:** 10.1186/s12938-021-00927-y

**Published:** 2022-04-12

**Authors:** Qingmin Wang, Yi Dong, Tianlei Xiao, Shiquan Zhang, Jinhua Yu, Leyin Li, Qi Zhang, Yuanyuan Wang, Yang Xiao, Wenping Wang

**Affiliations:** 1 Department of Electronic Engineering, Fudan University, Shanghai, 200433 China; 2 Department of Ultrasound, Zhongshan Hospital, Fudan University, 180 Fenglin Road, Shanghai, 200032 China; 3 Institute of Biomedical and Health Engineering Shenzhen Institutes of Advanced Technology, Chinese Academy of Sciences, 1068 Xueyuan Ave., Shenzhen, University Town, Shenzhen, 518055 China

**Keywords:** RF, Radiomics, HCC, PD-1, Ultrasound multifeature map

## Abstract

**Background:**

This study explored the feasibility of radiofrequency (RF)-based radiomics analysis techniques for the preoperative prediction of programmed cell death protein 1 (PD-1) in patients with hepatocellular carcinoma (HCC).

**Methods:**

The RF-based radiomics analysis method used ultrasound multifeature maps calculated from the RF signals of HCC patients, including direct energy attenuation (DEA) feature map, skewness of spectrum difference (SSD) feature map, and noncentrality parameter S of the Rician distribution (NRD) feature map. From each of the above ultrasound maps, 345 high-throughput radiomics features were extracted. Then, the useful radiomics features were selected by the sparse representation method and input into support vector machine (SVM) classifier for PD-1 prediction.

**Results and conclusion:**

Among all the RF-based prediction models and the ultrasound grayscale comparative model, the RF-based model using all of the three ultrasound feature maps had the highest prediction accuracy (ACC) and area under the curve (AUC), which were 92.5% and 94.23%, respectively. The method proposed in this paper is effective for the meaningful feature extraction of RF signals and can effectively predict PD-1 in patients with HCC.

**Supplementary Information:**

The online version contains supplementary material available at 10.1186/s12938-021-00927-y.

## Highlights

- We proposed RF-based radiomics analysis method by introducing three ultrasound features of direct energy attenuation (DEA), skewness of spectrum difference (SSD) and noncentrality parameter S of Rician distribution (NRD) as the feature extraction method from RF signals, investigated the effectiveness of RF-based radiomics analysis method in the immunocheckpoint prediction of programmed cell death protein 1 (PD-1), and validated the results with contrast testing of grayscale-based radiomics analysis method in this study. We also demonstrate a trend in prediction performance changes and its correlation with the number of ultrasound features.
- The results demonstrated that there were significant differences (*p* < 0.05) in radiomics scores between HCC patients with PD-1 and HCC patients without PD-1. RF-based radiomics analysis method performed well in PD-1 noninvasive preoperative prediction of HCC patients.
- In this study, the performance of RF-based radiomics analysis method was better than that of grayscale-based radiomics analysis method in the preoperative prediction of PD-1 in HCC patients. The AUC of DSNM, which was the RF-based radiomics analysis model with three ultrasound feature maps, reached 94.23% in the prediction of PD-1 cell protein in HCC patients.

**Supplementary Information:**

The online version contains supplementary material available at 10.1186/s12938-021-00927-y.

## Background

Hepatocellular carcinoma (HCC) is the second most common cause of cancer-related death [1]. To date, there is no clinical evidence that most of the adjuvant agents studied can improve survival in any stage of HCC [2]. In addition, the prognosis of HCC patients is generally poor. As the most common option for HCC patients, resection with curative intent or ablation is associated with 5-year recurrence rates as high as 75% [3]. Programmed cell death protein 1 (PD-1) could act as an indicative marker for the prognosis of HCC patients after surgical resection and may have a positive impact on the choice of treatment for HCC patients [4]. However, the current detection of PD-1 mainly depends on the immunohistochemical method with pathological tissue obtained by resection or puncture. There is a clear need to find a noninvasive, accurate preoperative PD-1 prediction technique for patients with HCC.

At present, anti-PD-1 antibody has been approved by the Food and Drug Administration (FDA) for the treatment of malignant melanoma and nonsmall cell lung cancer [5, 6]. A recent study found that PD-1 was highly expressed in the peripheral and intratumoral areas of HCC and could predict progression and postoperative recurrence [4]. However, PD-1 detection currently mainly depends on immunohistochemical staining of puncture or resected specimens. There are few studies on PD-1 prediction using other technologies. Although the trauma of puncture is small, the heterogeneity of the tumor and other reasons may cause inaccurate puncture results. Moreover, the puncture channel left in the liver tissue may damage the tumor microenvironment and stimulate the development and spread of the tumor. Therefore, it is urgent to develop a noninvasive and accurate method to predict PD-1 in HCC.

Ultrasonography is the first-line investigative technique for the surveillance of most diseases, as it has relatively low cost, noninvasive, and is widely available [7]. Surveillance of HCC with ultrasonography at 6-month intervals is recommended by the current guidelines [2, 8]. Radiomics is a new research method developed in the past decade [9, 10], that uses the computing power of computers to mine medical data in depth and extracts abundant tumor pathophysiology information that cannot be effectively found by the human eye [11, 12]. Therefore, applying radiomics analysis technology in ultrasound to deeply mine pathological information has great clinical development value. To date, it has performed well in breast cancer, HCC, liver fibrosis in chronic hepatitis B, nonsmall cell lung cancer, and so on [13–18]. This makes it possible and worth trying to research the performance of radiomics analysis techniques combined with the ultrasound data of HCC patients for PD-1 preoperative prediction.

The radiofrequency (RF) signal is the original ultrasonic signal without signal postprocessing (brightness compensation, envelope detection, depth compensation, dynamic range adjustment, etc.). It contains most of the acoustic information including attenuation, scattering, sound velocity, phase, and so on. In essence, it can provide more information than ultrasonic images [19]. In particular, large amount of high-frequency information before detection is suitable for algorithm compilation. However, the large amount of information from the RF signal is also accompanied by excessive noise interference. Even if an existing deep learning network with good performance is used, it is usually unable to create a successful model because of the large amount of noise and the abstract labels. At the same time, there is a lack of an effective feature extraction method to establish a diagnostic model directly using RF signals. Therefore, simplification can be considered by calculating as many physical parameters as possible. Then the intelligent model can be built on these physical parameter spectra, which is more likely to effectively realize the deep data mining of RF signals.

The attenuation [20], skewness [21], and Rician distribution [22] are the traditional characteristic parameters in ultrasound. In this study, direct energy attenuation (DEA), skewness of spectrum difference (SSD), and noncentrality parameter S of the Rician distribution (NRD) were used to compose three feature maps. We established an RF-based radiomics analysis method to extract radiomics features from ultrasound feature maps obtained by RF and realized the noninvasive prediction of PD-1 in HCC patients. Our aim was to investigate the value of the RF-based radiomics analysis algorithm in the preoperative prediction of PD-1 in patients with HCC. In summary, the contributions of this paper are as follows:

1. We proposed the RF-based radiomics analysis method by introducing the three ultrasound features of DEA, SSD, and NRD as the feature extraction method from RF signals, investigated the effectiveness of the RF-based radiomics analysis method in the immune checkpoint prediction of PD-1, and validated the results with contrast testing of the grayscale-based radiomics analysis method in this study. We also demonstrate a trend in prediction performance changes and its correlation with the number of ultrasound features.
2. The results demonstrated that there were significant differences (p < 0.05) in radiomics scores between HCC patients with PD-1 and HCC patients without PD-1. RF-based radiomics analysis method can realize the noninvasive preoperative prediction of PD-1 in HCC patients.
3. In this study, the performance of the RF-based radiomics analysis method was better than that of the grayscale-based radiomics analysis method in the preoperative prediction of PD-1 in HCC patients. The AUC of DSNM, which was the RF-based radiomics analysis model with three ultrasound feature maps, reached 94.23% in the prediction of PD-1 in HCC patients (Additional file 1).

## Results

### Ultrasound features results

In this study, we extracted multiple ultrasound parameters from RF signals, including DEA, SSD, and NRD. These three ultrasound parameters had varying degrees of positive roles in the preoperative prediction of PD-1. They were the basis of the ultrasound radiomics analysis method in this study. We compared the differences in the DEA, SSD, and NRD ultrasound feature parameters between patients with and without PD-1. ANOVA showed that there were no significant differences (*p* < 0.05) between patients with PD-1 and patients without PD-1 in DEA, SSD and NRD.

### Radiomics features results

Through feature extraction, 345 radiomics features were obtained from every ultrasound grayscale image and the DEA, SSD, and NRD ultrasound feature maps. A total of 345 radiomics features were extracted from every patient in the PD-1 prediction model based on ultrasound grayscale image (GM), 345 radiomics features were extracted from every patient in PD-1 prediction model based on ultrasound DEA feature map (DM), 690 radiomics features were extracted from every patient in PD-1 prediction model based on DEA and SSD feature maps (DSM), and 1035 radiomics features were extracted from every patient in PD-1 prediction model based on DEA, SSD, and NRD feature maps (DSNM).

The SRC coefficient represented the importance of the features relative to prediction. According to the above parameter, the high-throughput features of every model were sorted and the useful features were preliminarily selected. SRC feature selected part was the first time to reduce the feature dimension. After SRC feature selection, the feature numbers of GM, DM, DSM, and DSNM of the PD-1 prediction models were 241, 260, 432, and 564, respectively.

At this time, the feature number of every prediction model was still large. The preliminarily selected features were put into the SVM classifier to realize further feature dimension reduction. When each model used the SVM classifier for training, the first feature of its preliminarily selected features was put into the classifier for training, and the evaluation parameters of ACC, AUC, SPEC, and SENS after training were calculated. Then, the first two features of its preliminarily selected features were selected and put into the classifier for training, and the ACC and other evaluation parameters were also calculated. In this way, different numbers of features were extracted in turn to train the SVM classifier, and the corresponding evaluation parameters were recorded. Finally, the best result was selected through the saved evaluation parameters. The number of features put into the classifier corresponding to the best result was the final feature dimension of this model after the second dimension reduction. These features were also the final features of every PD-1 classification prediction model. In this way, the final feature dimensions of the PD-1 prediction models of GM, DM, DSM, and DSNM were 33, 13, 13, and 10, respectively.

### Prediction model results

The performance of the GM PD-1 prediction model and other RF-based PD-1 prediction models are shown in Table 1. As a contrast experiment, GM used grayscale images, and its AUC and ACC reached 80.77% and 80.00%, respectively. However, the SENS of this model was low, only 57.14%. The performance outcomes of the RF-based DM, DSM, and DSNM models were better than that of GM.

**Table 1:** Diagnostic performance of GM, DM, DSM, and DSNM for PD-1 classification

Model type	AUC (%)	ACC (%)	SENS (%)	SPEC (%)
GM	80.77	80.00	57.14	92.31
DM	83.52	85.00	71.43	92.31
DSM	88.46	87.50	78.57	92.31
DSNM	94.23	92.5	92.86	92.31

AUC, area under the receiver operating characteristic curve; ACC, accuracy; SENS, sensitivity; SPEC, specificity; GM, PD-1 prediction model based on ultrasound grayscale image; DM, PD-1 prediction model based on DEA feature map of RF signals; DSM, PD-1 prediction model based on DEA and SSD feature maps; DSNM, PD-1 prediction model based on DEA, SSD, and NRD feature maps.

The ACC, AUC, and SENS of DSNM were the largest among the three RF-based prediction models. The AUC of DSNM was 94.23% (95% confidence interval [CI] 0.820 to 0.991). The AUCs of DSM and DM reached 88.46% (CI 0.744 to 0.964) and 83.52% (CI 0.684 to 0.933), respectively. With the increase in the number of RF-based ultrasound feature maps, the performance of the PD-1 prediction models for HCC patients gradually improved.

**Fig. 1:**
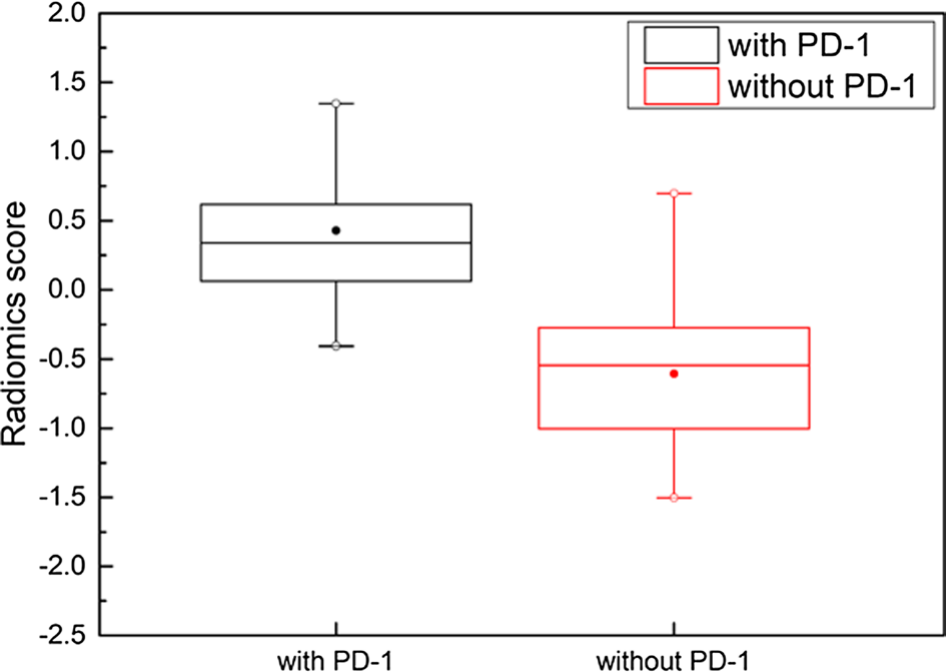
Boxplot of the radiomics scores of DSNM PD-1 prediction model for HCC patients with and without PD-1

The SVM classifier in the PD-1 prediction model was used to calculate the radiomics score of each HCC patient. The model can predict the presence of PD-1 in HCC patients based on the radiomics score. Figure 1 shows a boxplot of the radiomics scores of the DSNM PD-1 prediction model for HCC patients with and without PD-1. ANOVA showed that there was a significant difference (*p* < 0.05) between patients with PD-1 and patients without PD-1.

**Fig. 2:**
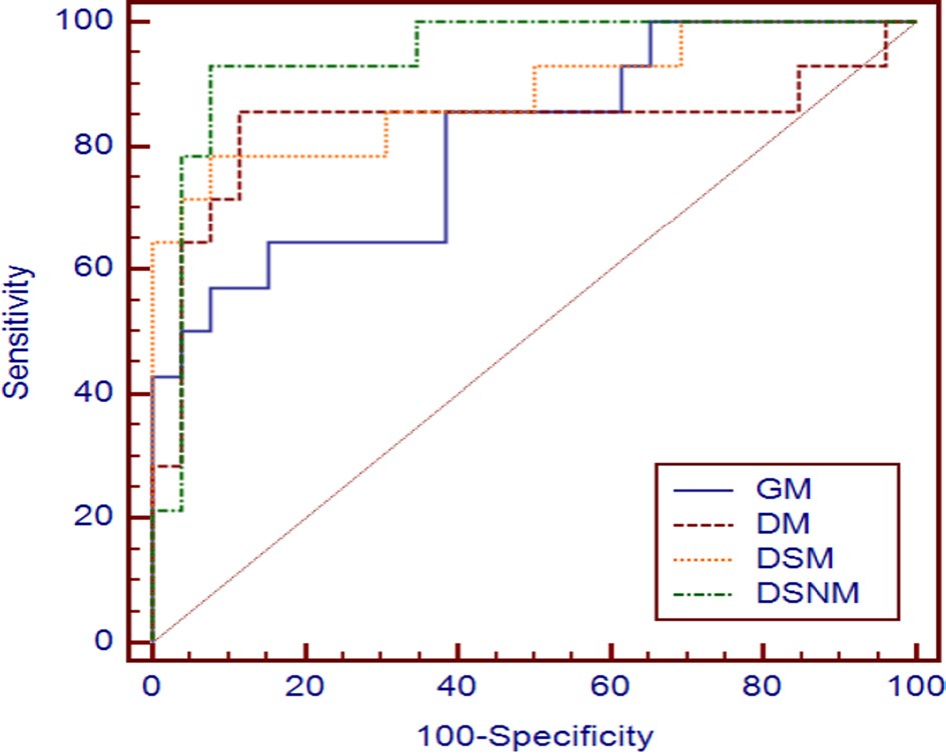
Comparison of the ROC curves of DSNM, DSM, DM, and GM PD-1 prediction models

The ROC curves of the grayscale-based and RF-based PD-1 prediction models are shown in Fig. 2. The total areas under the ROC curve can also represent the AUCs of the PD-1 prediction models. The area under the ROC curve of DSNM was 0.94 ± 0.04, which was the largest of the four PD-1 prediction models of HCC patients.

**Fig. 3:**
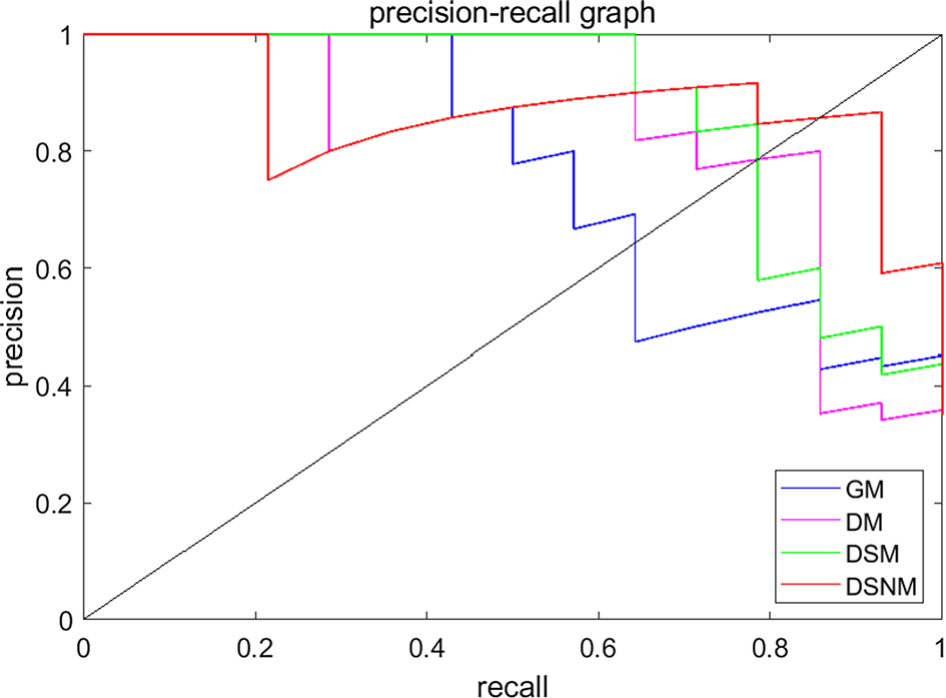
Precision-recall curves of the GM, DM, DSM, and DSNM PD-1 prediction models

The PRCs of DSNM, DSM, DM, and GM are shown in Fig. 3. The break-even point (BEP) in the PRC is the basis for judging the performance of the PD-1 prediction models. The BEP is the value when the precision of the model is equal to the recall. The BEP of the DSNM model in Fig. 3 is the intersection of the red curve and the diagonal. Its value is larger than that at the intersections of the other three models and the diagonal. This indicates that the DSNM based on three ultrasound feature maps calculated from RF signals has the best performance of all four models in predicting the PD-1 in HCC patients.

## Discussion

This study demonstrated that the combination of ultrasound multifeature maps of RF signals and radiomics analysis was highly effective in predicting PD-1 of HCC patients. This is the first report of the preoperative prediction of PD-1 in HCC patients by using radiomics technology based on ultrasound multifeature maps of RF data. Three prediction models based on RF signals and one prediction model based on grayscale images were established in this study. The results showed that the three prediction models based on RF all performed better than the model based on grayscale images in predicting PD-1 in HCC. Among the above three RF-based prediction models, the DSNM model using DEA, SSD, and NRD, which are three kinds of ultrasound feature maps, performed best, with an AUC of 0.94 ± 0.04. The texture features and wavelet texture features extracted from the three ultrasound feature maps in DSNM model had the best accuracy for PD-1 preoperative prediction. Thus, RF-based ultrasound multifeature map radiomics analysis had a positive effect on the prediction of this immune checkpoint inhibitor. In this study, the grayscale-based radiomics analysis contrast test and LOOCV ensured the correctness of the DSNM prediction method.

In radiomics, changes in tumor microproteins, molecules, and genes are closely related to changes in macro medical imaging. Many studies on radiomics have confirmed this phenomenon in CT [9], PET [23, 24], and MRI [25–28] images. From the above images, researchers extracted features related to the proteins and genes studied. At the same time, radiomics technology based on the above imaging techniques provides assistance for disease diagnosis, clinical decision-making, and prognosis. At present, the development of radiomics in ultrasound is in the initial stage. Since ultrasound is noninvasive, does not require radiation, is inexpensive, and is widely used, the development space of radiomics in ultrasound is very large. Qiao et al. used B-mode ultrasound images to identify benign and malignant breast tumors [14]. Zhang et al. [13] extracted high-throughput radiomics features from ultrasound elastic images for the diagnosis of breast tumors. These are examples of ultrasound image-based radiomics methods. In this study, we used the method similar to the above to establish the GM model to predict the immunosuppressive molecules PD-1 of HCC patients. The AUC of grayscale image-based GM model was 80.77% in prediction of the cell surface receptor PD-1. This grayscale image-based model was established to compare with the RF-based models.

Grayscale images are most widely used in ultrasound radiomics. A recent study by Biermann et al. proved that grayscale image-based radiomics model slightly outperformed ACR scoring by the less experienced radiologists in the classification of thyroid nodules [29]. The combination of multiple ultrasound images is one of the development trends of ultrasound radiomics. Xue et al. [30] confirmed that the combination of grayscale images and elasticity images in the transfer learning radiomics model was the most accurate prediction model in liver fibrosis grading (AUCs are 0.950, 0.932, and 0.930 for classifying S4, ≥ S3, and ≥ S2, respectively). No matter grayscale images or elasticity images, they are all calculated from RF signals. RF signal itself contains more information than these images. However, an effective feature extraction method of RF to establish the diagnostic model directly is lacking. The study of Wei et al. mentioned that using a single modality of RF signals for the liver fibrosis stage, the highest accuracy of the verification set was only 0.77 [31]. How to extract as much useful information as possible and improve the utilization efficiency of RF signals is worthy of exploration. Wei et al. chose the method of combing multiparametric features, including ultrasound grayscale images, RF signals, and contrast-enhanced micro-flow images, and the highest classification accuracy was 0.12 higher than that of the single modality of RF for the liver fibrosis stage [31]. In our study, we proposed to calculate three kinds of ultrasonic features from RF signals, including DEA, SSD, and NRD, and then extract effective features for the second time with radiomics method, so as to simplify and get useful information from RF signals as much as possible. The results showed that the performances of PD-1 prediction models using DEA, SSD, and NRD ultrasound feature maps were generally better than that of using only ultrasound grayscale images. Moreover, the more the ultrasound feature maps used, the better the PD-1 prediction performance.

As shown in Figs. 2 and 3, the model using DEA, SSD, and NRD ultrasound feature maps simultaneously showed the best performance of all the RF-based models using ultrasound feature maps, with an AUC of 94.23%. The attenuation coefficient of biological tissue is closely related to its tissue properties and structural characteristics. The differences caused by high PD-1 expression in disease tissues may vary, such as sound velocity, acoustic impedance, and acoustic attenuation coefficients. In this study, the DEA coefficient was used as an ultrasound feature to establish a DM preoperative PD-1 prediction model. The AUC of this model reached 83.52%. Studies on the frequency domain analysis of HCC mainly focus on the internal blood flow spectrum. The SSD feature map is a spectrum feature and reflects the frequency domain characteristics of the RF signals of the whole ROI of HCC lesions. In fact, the addition of the SSD ultrasound feature map to the DM model to form the DSM model improved the PD-1 prediction results. Statistical distribution models, such as Rician distribution, are still lacking in the detection of the PD-1 receptor. High-throughput feature extraction in radiomics technology is helpful to distinguish the ultrasound imaging changes caused by the accumulation of the tumor PD-1 receptor. The combination of radiomics analysis technology and ultrasound feature maps composed of the ultrasound feature parameters DEA, SSD, and NRD achieved 92.5% ACC in the DSNM model for PD-1 prediction in HCC patients. With the increase in the types of ultrasound feature maps, the predictive performance of the model increased. This results suggest that more ultrasound features should be extracted from RF signals, combined with high-throughput imaging features, to fully develop the application of ultrasound at the molecular and protein levels.

ANOVA showed that there were no significant differences in DEA, SSD, and NRD (*p* < 0.05) between patients with PD-1 and patients without PD-1. However, the radiomics scores of HCC patients based on the DSNM PD-1 prediction model in the boxplot of Fig. 1 shows that there were significant differences between HCC patients with and without PD-1. This explains why the numerical value of ultrasound feature maps calculated from RF in this research cannot directly and effectively predict the presence of PD-1 in HCC patients. After radiomics processing, the extracted texture features and wavelet-based texture features achieved a prediction accuracy of more than 85% in all three prediction models of DM, DSM, and DSNM. In addition to the traditional numerical value of ultrasound feature parameters, the numerical distribution characteristics and texture features of the ultrasound feature parameters themselves also provide useful clues worthy of study. It is suggested that the combination of radiomics processing methods can further mine the information contained in ultrasound RF signals and expand the application scope of ultrasound diagnosis. In this paper, an effective feature extraction method from RF signals for PD-1 prediction in HCC patients was established.

Texture features are one of the most widely utilized features in radiomics and perform well in terms of benign and malignant identification [32], protein [26], gene prediction [33, 34], and molecular typing [35]. Pham et al. [32] extracted two kinds of texture features from CT images of lung cancer patients to differentiate of mediastinal lymph nodes. Dang et al. [26] extracted texture features from MRI images to predict the tumor suppressor protein p53 in head and neck squamous cell carcinoma, with an accuracy of 0.813. Yang et al. extracted 97 texture features from MRI images and combined them with a random forest classifier to carry out molecular typing classification and survival prediction of glioma [35]. In this study, we extracted 345, 345, 690, and 1035 texture features and wavelet-based texture features from the GM, DM, DSM, and DSNM models, respectively. These texture features were simplified after SRC feature selection and secondary dimension reduction in SVM classification, which supports the successful prediction of PD-1. The preoperative prediction ACCs of the cell receptor PD-1 for HCC patients in the GM, DM, DSM, and DSNM models were 80%, 85%, 87.5%, and 92.5%, respectively. Texture features, as important visual features that are difficult to describe in detail for doctors, still have significant advantages in effective feature extraction from RF-based ultrasonic feature maps.

However, some limitations should be noted in this study. First, only 40 available patients were screened from 129 patients. Independent testing is difficult for small sample sizes. For this reason, this study designed a grayscale image-based comparative test to verify the prediction accuracy of the RF signal-based model while using the LOOCV method. Second, research at a single center cannot verify the generalization ability of the model. The next step of the research will be to achieve multicenter research results. Third, the current prediction results of this study can only predict PD-1 negative and positive results for HCC patients. Tumor heterogeneity makes it difficult to represent the nature of the whole pathological tissue. This makes the results of immunohistochemistry and tumor molecular typing less reliable for the development of the follow-up treatment plan. It also increases the uncertainty of prognosis. Studying how to predict the distribution, proportion, and area of PD-1 in the ROI may provide strong technical support for the selection of puncture sites. The treatment plan and prognosis may be more accurate with this information.

## Conclusion

In conclusion, we propose an RF-based radiomics analysis method for predicting PD-1 in HCC patients. The DSNM model using RF-based ultrasound multifeature maps and the radiomics analysis method has the best performance of all four models and is expected to become a robust method for the noninvasive and fast preoperative prediction of PD-1 in HCC patients. Although the RF signal contains more information than the traditional grayscale image, an effective feature extraction method to establish the diagnostic model directly is lacking. The application of the ultrasound multifeature map extraction method and radiomics feature extraction effectively improves the utilization value of ultrasound RF signals and provides deeper diagnostic and treatment information from ultrasound. RF signals can provide richer diagnostic information than ultrasound images. The proposed method can provide a valuable reference for the combination of ultrasound and radiomics analysis and facilitate the development of more accurate algorithms and clinical diagnostic aids.

## Materials and methods

### Patients

From January 2018 to December 2018, we enrolled 129 liver cancer patients preoperatively diagnosed with HCC in a designated institution. Finally, 40 eligible patients (33 men and 7 women; age range: 23–80 years; mean: 55 ± 12 years) were selected for this study. The inclusion criteria were (1) patients with HCC confirmed by pathological examination and operation; (2) patients with a solitary tumor; (3) patients who underwent preoperative grayscale ultrasound examinations within 1 week before surgery and had useful RF data; and (4) patients with confirmation by histopathological examination and PD-1 evaluation.

The exclusion criteria included (1) patients without HCC confirmed by pathological examination; (2) patients with preoperative biopsy or adjuvant therapy; (3) patients with an incomplete or not clearly visible HCC lesion area reconstructed by RF data; and (4) patients without histopathological examination and PD-1 evaluation results.

PD-1 evaluation was performed by two pathologists with at least 10 years of experience in hepatopathology reviewing all the specimen slices. Both investigators were blinded to the clinical and imaging information of the patients.

### Ultrasound data acquisition

All examinations, including conventional ultrasound and RF ultrasound, were performed on an EPIQ-7 ultrasound system (Philips Medical Systems, Amsterdam, Holland). A C5-1 curved transducer with frequencies of 1–5 MHz (Philips Medical Systems, Amsterdam, Holland) was used for data acquisition, including ultrasound grayscale images and corresponding RF data.

All patients fasted for at least 8 h before ultrasound examinations. Then, the grayscale ultrasound features of the hepatic lesions were assessed according to a standardized protocol: number of lesions (solitary or multiple), size of the lesion (mm), and echogenicity (hyperechoic, isoechoic, hypoechoic, or mixed compared to surrounding liver tissue). Ultrasound grayscale examinations were performed by a single experienced radiologist (with more than 18 years of experience in ultrasound of the liver).

### RF data processing

**Fig. 4:**
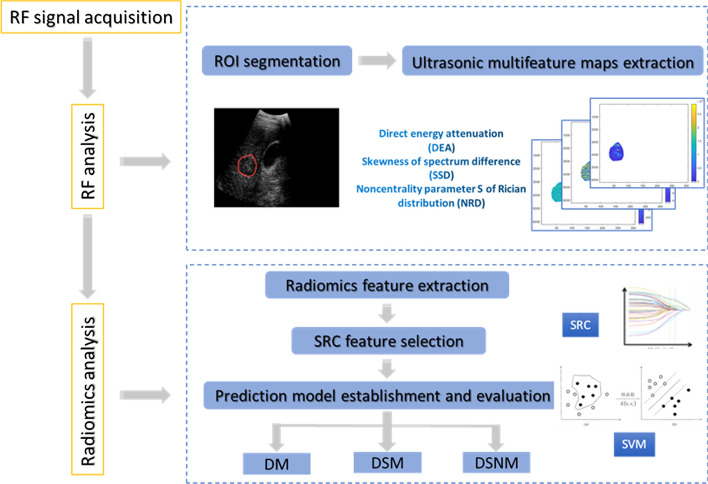
Experimental flow diagram of the RF-based radiomics analysis method

For the RF data obtained in this study, the specific RF data processing flow, which we call the RF-based radiomics analysis method, is shown in Fig. 4. We first conducted RF analysis to extract ultrasound multifeature maps. Then, combined with the widely used radiomics analysis method, high-throughput radiomics features were extracted, selected, and used to build effective PD-1 classification prediction models.

#### Ultrasound multifeature map extraction

**Fig. 5:**
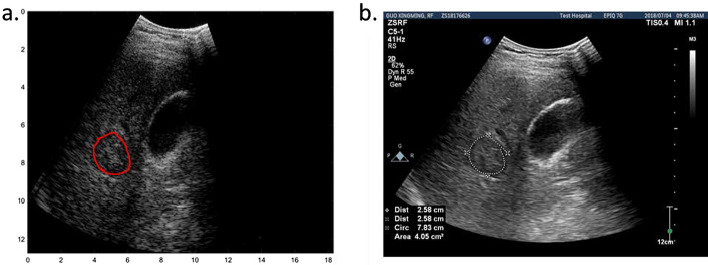
**a** B-mode image of a patient reconstructed by RF data. **b** B-mode image saved during data acquisition in the hospital with a white dotted circle marked by the doctor during diagnosis

The extraction of ultrasound multifeature maps was a unique design in this study and effectively improved the performance of ultrasound RF data in making classifications and predictions of the PD-1 protein level. This will be mentioned later in the analysis of the experimental results. To improve the calculation efficiency of ultrasound multifeature map extraction, we extracted the RF data of the region of interest (ROI) and used these data instead of the whole echo RF dataset to calculate the multifeature maps. Then, smooth filtering, Hilbert transformation, logarithmic compression, sector transformation, and other processing method were carried out on the RF data to achieve B-mode reconstruction, as shown in Fig. 5a. By referring to the lesion location marked with a white dotted circle by the doctor in the corresponding grayscale image saved during data acquisition, as shown in Fig. 5b, we can determine and segment the ROI, which is shown in Fig. 2a with a red circle, to obtain the RF data of the ROI.

**Fig. 6:**
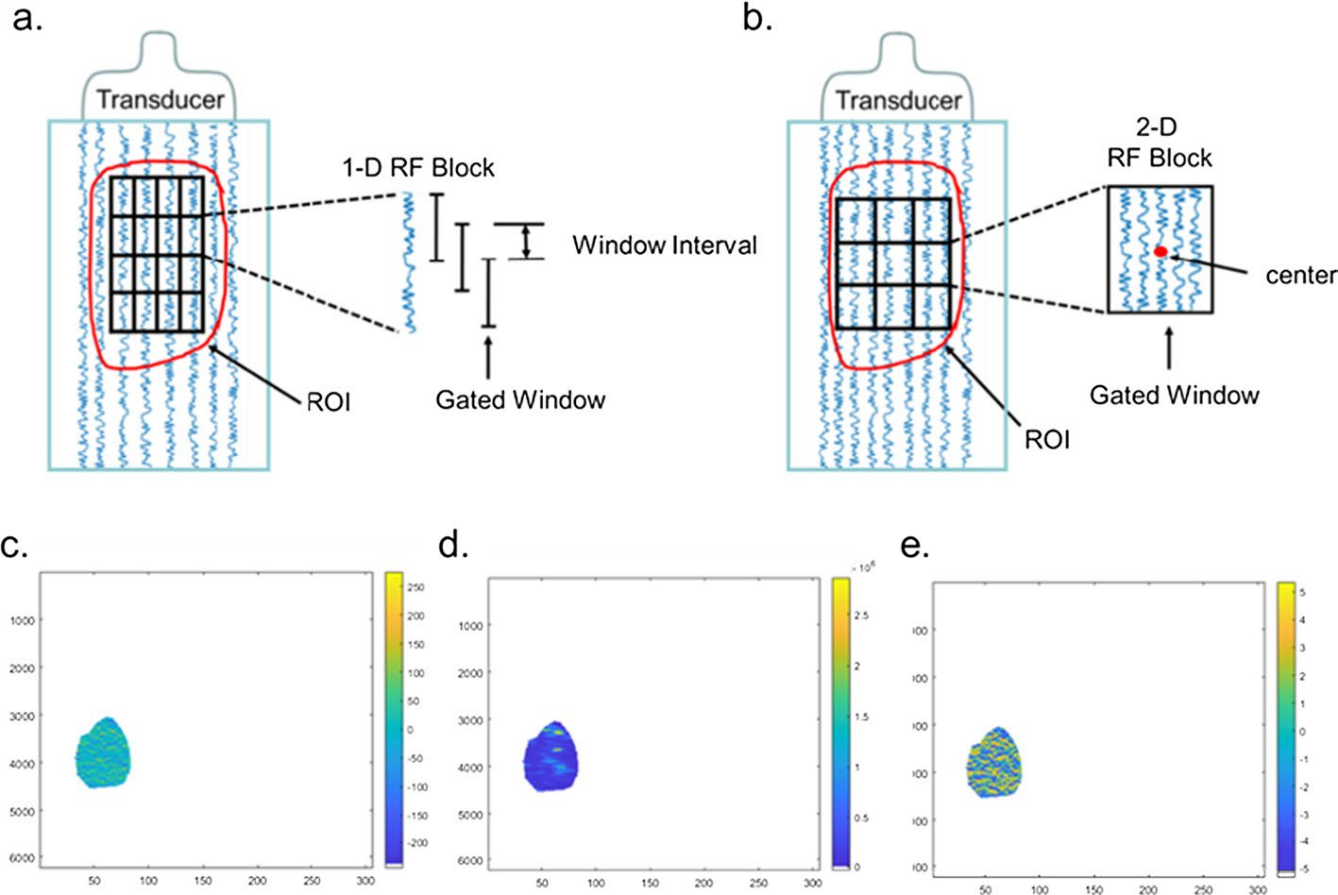
Schematic diagram of the extraction method of the **a** 1-D RF data block and **b** 2-D RF data block of the ROI in ultrasound feature map calculation. **c** Direct energy attenuation (DEA) ultrasound feature map. **d** Skewness of spectrum difference (SSD) ultrasound feature map. **e** Noncentrality parameter S of the Rician distribution (NRD) ultrasound feature map

The RF data of the ROI were used to calculate the three feature parameters of direct energy attenuation (DEA), skewness of spectrum difference (SSD), and noncentrality parameter S of Rician distribution (NRD), which were composed of the corresponding DEA , SSD, and NRD ultrasound feature maps, as shown in Fig. 6. There are many kinds of ultrasound features that can characterize RF signals, including time domain features, frequency domain features, geometric features, and statistical features. When there is PD-1 protein in the liver of HCC patients, the microscopic scattering differences may manifest in the time domain, frequency domain, and so on. In this study, the DEA, SSD, and NRD ultrasound features we chose belong to the time domain feature, frequency domain feature, and statistical feature, respectively.

DEA refers to the direct energy attenuation of ultrasound propagation in the medium. As shown in Fig. 3a, two 1-D RF data blocks with a gated window length of 64 and window interval of 16 were extracted by moving down one data point each time. Fourier transforms were applied to obtain the average energy ${\mathrm{E}}_{0}$ of the 1-D RF data block directly above and ${\mathrm{E}}_{1}$ of the 1-D RF data block directly below. Formula (1) was used to calculate the DEA coefficient in this study:

1 $${\text{DEA}} = 10*\log \left( {E_{0} /E_{1} } \right)/\left( {D*C/f_{S} /2} \right),$$

where D represents the gated window length, which is 64 data points; C equals 1540 m/s, which represents the sound speed of the ultrasound wave in tissue; and ${f}_{S}$ represents the sample rate, which is 32 MHz. The DEA value calculated is that of the point (window length + window interval)/2 on the 1-D RF data block directly above.

SSD represents the skewness of the spectrum difference of the ultrasound RF echo signal. Skewness is calculated by the following formula (2):

2 $$S = \frac{{\frac{1}{n}\mathop \sum \nolimits_{i = 1}^{n} \left( {x_{i} - \overline{x}} \right)^{3} }}{{\left( {\frac{1}{n}\mathop \sum \nolimits_{i = 1}^{n} \left( {x_{i} - \overline{x}} \right)^{2} } \right)^{\frac{3}{2}} }},$$

where *S* is the skewness (dimensionless); the letter ‘*i*’ is the *i*-th number; $\overline{X }$ is the average of the samples; *n* is the number of samples; and ${x}_{i}$ is the *i*-th sample value.

A schematic diagram how the 1-D RF data block of the ROI was extracted during the SSD calculation is shown in Fig. 3a. Two 1-D RF data blocks with window lengths of 64 were extracted by moving down one data point each time. The window interval between the two RF data blocks was 40. Fourier transform was applied on the two 1-D RF data blocks. Then make a subtraction between the two blocks obtained by Fourier transform, and the skewness of the data after subtraction was calculated by using the function of ‘skewness’ in the MATLAB toolbox. The calculated SSD value is that of the point (window length + window interval)/2 on 1-D RF data block located directly above.

NRD is the noncentrality parameter *S* of the Rician distribution. The Rician distribution has the following density formula (3):

3 $$p\left( R \right) = I_{0} \left( {\frac{xs}{{\sigma^{2} }}} \right)\frac{x}{{\sigma^{2} }}^{{ - \left( {\frac{{x^{2} + s^{2} }}{{2\sigma^{2} }}} \right)}} ,$$

with noncentrality parameter s $\ge$ 0 and scale parameter $\sigma$ > 0, for *x* > 0, which is the sample value. ${\mathrm{I}}_{0}$ is the zero-order modified Bessel function of the first kind. In this study, the noncentrality parameter *S* of the midpoint of each 2-D RF data block was calculated by the function of ‘fitdist’ in the MATLAB toolbox. The 2-D data block selection method is shown in Fig. 3b. The size of each selected 2-D data block was 37 × 8. The *S* value of the Rician distribution of the selected data block was calculated as the NRD value of the midpoint of the current 2-D data block. Then, the gated windows were moved down one data point by one data point, and all the NRD values of each point in the ROI could be calculated.

The above three ultrasound feature parameters are widely used and representative and include most of the features of RF signals. These parameters compose the three ultrasound feature maps of the DEA, SSD, and NRD. The three ultrasound feature maps are shown in Fig. 3c–e.

#### Radiomics feature extraction and selection

Radiomics feature extraction was used to extract texture features and wavelet-based texture features from the three ultrasound feature maps of DEA, SSD, and NRD. Texture is ubiquitous in medical images and is an important visual clue of imaging doctors for diagnose. Each ultrasound feature map was extracted 69 texture features, including histogram features, gray-level co-occurrence matrix (GLCM) features, gray-level run-length matrix (GLRLM) features, grey-level size-zone matrix (GLSZM) features, and neighborhood gray-tone difference matrix (NGTDM) features.

In addition, texture features often show multiscale characteristics. Wavelet transform, as a multiscale analysis tool, can adaptively obtain the effective signals of different frequency components of the original image. We used wavelet transform to obtain 4 different frequency components of each ultrasound feature map and extracted the above 69 texture features from these maps, respectively. Then, another 276 wavelet-based texture features of each ultrasound feature map were obtained.

The detailed radiomics features are shown in Table 2. A total of 345 radiomics features were extracted from each ultrasound feature map and its 4 frequency components.

**Table 2:** Detailed radiomics features extracted from each ultrasound feature map and its 4 frequency components

Kinds of high-throughput radiomics features	Texture features from each ultrasound feature map	Wavelet-based texture features from frequency component 1	Wavelet-based texture features from frequency component 2	Wavelet-based texture features from frequency component 3	Wavelet-based texture features from frequency component 4
Histogram	16	16	16	16	16
GLCM	13	13	13	13	13
GLRLM	22	22	22	22	22
GLSZM	13	13	13	13	13
NGTDM	5	5	5	5	5
Total	69	276			

GLCM (gray-level co-occurrence matrix), GLRLM (gray-level run-length matrix), GLSZM (grey-level size-zone matrix), and NGTDM (neighborhood gray-tone difference matrix)

The method of feature selection was based on sparse representation coefficient (SRC), which was proposed by Li [36]. The hypothesis of sparse representation was that all signals in the world are sparse and can be expressed sparsely, that is, they can be expressed linearly by finite features. The basic principle of sparse representation can be described by the following formula (4):

4 $$\mathrm{s}=\mathrm{\varnothing \beta }.$$

Suppose ${\varphi }_{i}\epsilon {R}^{N},i=\mathrm{1,2},\dots$, M is the base signal (atom) of the N*1 dimension, $\mathrm{\varnothing }=[{\varphi }_{1},{\varphi }_{2,\dots }{\varphi }_{M}]$ is the matrix composed of M base signals, which is called the dictionary, where M > N, β is the coefficient vector of the M*1 dimension, and s is the target signal of N*1. The goal is to select as few atoms as possible in $\mathrm{\varnothing }$ to make $\mathrm{\varnothing \beta }=\mathrm{s}$ tenable, that is, to find a β to make $\mathrm{\varnothing \beta }=\mathrm{s}$ tenable, while the number of nonzero elements in $\upbeta$ is as small as possible. $\upbeta$ can be solved by the OMP algorithm [37]. The elements in β are called the SRCs. The SRC value can reflect the importance of these features. All the radiomics features extracted were arranged in descending order by SRC according to their importance to the label in selecting the required number of useful features.

#### Establishment and evaluation of the prediction model

The currently widely used SVM classifier with excellent performance was used to build the PD-1 prediction model. This classifier can deal with the classification problem by the kernel method when the relationship between class labels and radiomics features is nonlinear. The kernel function method maps the linearly inseparable features of low-dimensional space to high-dimensional space through feature transformation to obtain the optimal separation hyperplane and realize linear classification. The Gaussian radial basis function was the preferred kernel function in this experiment. This function can map the original feature vector to the infinite dimension space to find the optimal hyperplane. There are few adjustable parameters using the Gaussian radial basis function, and only $\upgamma$ and the penalty parameter $\mathrm{C}$ can be changed. By using cross-validation to find the appropriate parameters $\mathrm{C}$ and $\upgamma$, the classifier can correctly predict the test set data. In this experiment, $\mathrm{C}$ was 0.8 and $\upgamma$ was 1. The SVM software package used in this experiment was the ‘libsvm’ toolkit designed by Lin Zhiren, Associate Professor of Taiwan University.

**Fig. 7:**
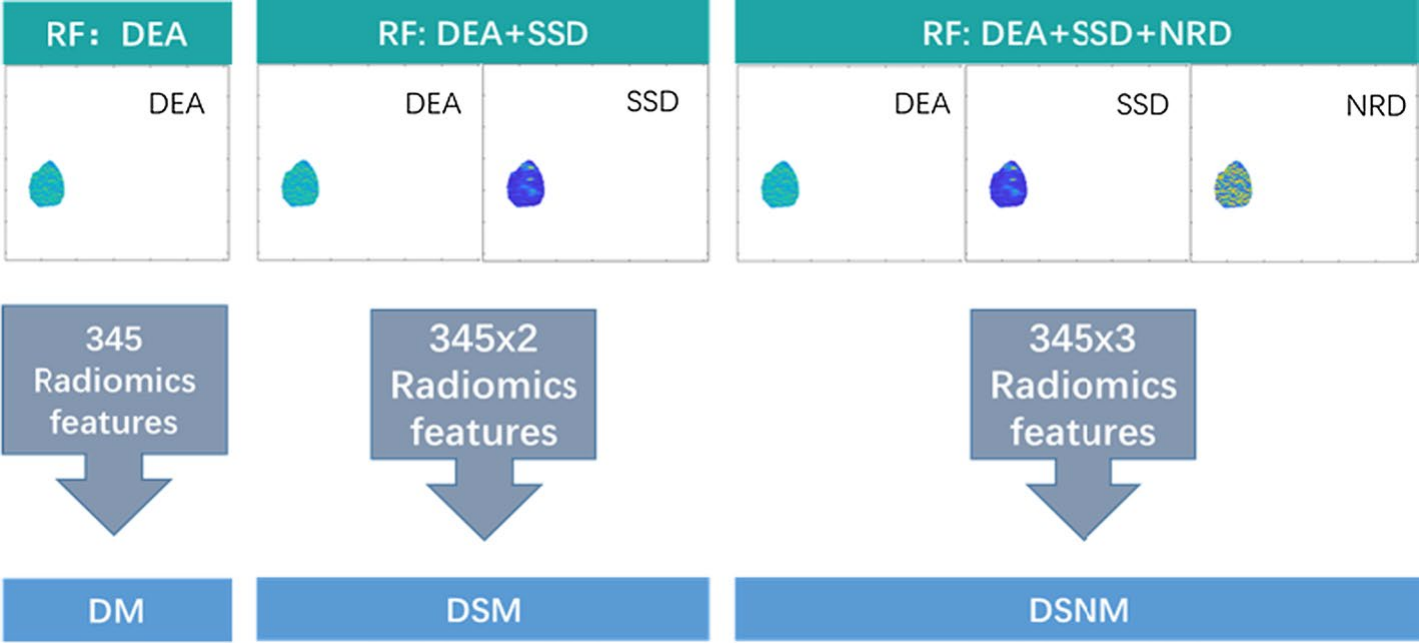
Three PD-1 radiomics prediction models based on RF included a PD-1 prediction model that used the DEA feature map (DM), a PD-1 prediction model that used the DEA and SSD feature maps (DSM), and a PD-1 prediction model that used the DEA, SSD, and NRD feature maps (DSNM)

Three PD-1 radiomics prediction models based on RF were established using SVM, including a PD-1 prediction model that used the DEA feature map (DM), a PD-1 prediction model that used the DEA and SSD feature maps (DSM) and a PD-1 prediction model that used the DEA, SSD, and NRD feature maps (DSNM), which are shown in Fig. 7.

### Grayscale image compression test

Ultrasound grayscale image-based radiomics analysis is a relatively traditional method and was compared with RF-based radiomics analysis methods in terms of the performance of PD-1 prediction in HCC patients. After segmenting the ROI of the grayscale image, the same radiomics analysis processing steps as that of RF data were carried out on the grayscale image. Ultrasound grayscale image-based radiomics analysis compression tests directly extracted 345 radiomics features from ROIs of the grayscale images. They were also selected by the SRC and were used to build the PD-1 prediction model based on grayscale images, which was called GM.

### Statistical analysis

The performance of the prediction model was evaluated by the LOOCV statistical analysis method. The Tukey’s test, in conjunction with analysis of variance (ANOVA), was used to test the significance between any two pairs of the three ultrasound features. Receiver operating characteristic (ROC) curves and precision-recall curves (PRCs) were employed to show the overall performance of the model. Other assessment indicators included the area under the ROC curve (AUC), accuracy (ACC), sensitivity (SENS), and specificity (SPEC). Descriptive statistics are summarized as mean ± SD.

## Supplementary Information

**Additional file 1.** Radiomics scores of the patients in all models.

## Data Availability

The datasets used and analyzed during the current study are available from the corresponding author on reasonable request.

## Abbreviations

RF: Radiofrequency
PD-1: Programmed cell death protein 1
HCC: Hepatocellular carcinoma
ROI: Region of interest
SR: Sparse representation
SVM: Support vector machine
LOOCV: Leave-one-out cross-validation
DEA: Direct energy attenuation
NRD: Noncentrality parameter S of Rician distribution
SSD: Skewness of spectrum difference
GM: Programmed cell death protein 1 prediction model based on ultrasound grayscale image
DM: Programmed cell death protein 1 prediction model based on direct energy attenuation
DSM: Programmed cell death protein 1 prediction model based on direct energy attenuation and Skewness of spectrum difference
DSNM: Programmed cell death protein 1 prediction model based on direct energy attenuation, noncentrality parameter S of Rician distribution and skewness of spectrum difference
AUC: Area under the curve
ACC: Accuracy
SENS: Sensitivity
SPEC: Specificity
MRI: Magnetic resonance imaging
CT: Computed tomography
FFT: Fast Fourier transform
GLCM: Gray-Level Co-Occurrence Matrix
GLRLM: Gray-Level Run-Length Matrix
GLSZM: Grey-Level Size-Zone Matrix
NGTDM: Neighborhood Gray-Tone Difference Matrix
ROC: Receiver operating characteristic curve
PRC: Precision-recall curve
BEP: Break-even point
